# A New GNSS Spoofing Signal Power Control Algorithm for Receiver Sensors in Acquisition Phase and Subsequent Control

**DOI:** 10.3390/s22176588

**Published:** 2022-08-31

**Authors:** Yangjun Gao, Guangyun Li

**Affiliations:** 1 State Key Laboratory of Geo-Information Engineering, Xi’an 710054, China; 2 College of Geospatial Information, PLA Strategic Support Force Information Engineering University, Zhengzhou 450001, China

**Keywords:** GNSS spoofing, receiver sensors, spoofing signal, power control algorithm, signal acquisition

## Abstract

Satellite navigation spoofing technology has become a hotspot of interference technology research because of its significant threat and high concealment. In a spoofing scenario, suppressive interference is typically used to ensure that the target receiver sensor is in the unlocked and reacquisition state, and then spoofing is implemented. This method has a high feasibility, and the power of the spoofing signal affects the concealment and efficiency of spoofing. Currently, there is limited research involving the GNSS spoofing signal power control. Moreover, there is no systematic complete power control scheme, most of which is limited to qualitative or simulation, and the actual application effect is still unclear. Therefore, a new GNSS spoofing signal power control algorithm under the power constraints of the receiver sensor in the acquisition phase and the subsequent control is proposed. The experimental platform is designed to prove that compared with the conventional spoofing signal high power control algorithm, the new GNSS spoofing signal power control algorithm shortens Doppler frequency fluctuation time by 72.2% and reduces the range by 75.9%. The carrier-to-noise ratio of the received signal is less than the threshold of the receiver sensor, and the range of three-dimensional coordinates of Earth-Centered, Earth-Fixed (ECEF) is significantly reduced during the spoofing signal taking over receiver sensor, this shows that the new design of the GNSS spoofing signal power control algorithm can make spoofing behavior more hidden, and it will make it more difficult for the target receiver sensor to detect spoofing behavior. The designed algorithm can take over the receiver sensor stealthily with the help of suppressing interference and then pull the bias positioning results, which has good feasibility and effectiveness.

## 1. Introduction

As the application of the satellite navigation system has penetrated into all aspects of social life and military applications, the navigation terminal may receive incorrect timing and positioning results owing to spoofing signals, which may lead to catastrophic consequences. Spoofing may gradually become a severe threat to satellite navigation systems [1]. Since the US Transportation Department first raised concerns regarding spoofing in satellite navigation in 2001, spoofing has been attracting increasing attention from several countries, especially the military, and has gradually become a hotspot in satellite navigation interference technology research [2].

Spoofing refers to the interference technology, wherein the interference source generates a spoofing signal that is highly similar to the authentic satellite navigation signal or forwards the authentic signal, causing the target receiver sensor to misinterpret the spoofing signal as the authentic satellite navigation signal for acquiring and tracking, which results in the receiver sensor outputting error messages or without information. Spoofing is more destructive and threatening than other forms of interference [3].

Regardless of whether the spoofing is spoofing on a target receiver in the signal acquisition phase or the tracking phase, the control of the spoofing signal power is an important problem to be studied. When a receiver is in the acquisition phase, the spoofing signal can effectively spoof the receiver by generating multiple false correlation peaks and increasing the noise floor [4]. During the signal acquisition phase, the receiver searches within the two-dimensional space of the Doppler shift and the code phase, and calculates the signal parameters at the highest correlation peak above the priori decision threshold [5]. A spoofing signal produces a higher power correlation peak in the search domain by generating a higher power signal. At this time, a receiver is easily locked at the peak of the spoofing signal, thereby affecting the positioning result of the receiver [6].

In an actual scenario, it is better to use the method of suppressing interference to make receiver lose lock, and then implement the method of spoofing [7]. When the receiver is in cold start or loss lock and reacquisition (loss of lock caused by natural environment or suppressed interference), a spoofing signal present in the environment may be and acquired by the receiver. The method of entering the receiver through the acquisition phase is simple and effective. However, for a receiver with a certain spoofing detection capability, after it is successfully pulled into the spoofing signal, to effectively implement persistent spoofing, it is necessary to control the power without being noticed by the receiver. For example, in a normal environment, the noise floor of the receiver is relatively stable; however, when a spoofing signal invades, there is a cross-correlation interference between the spoofing signal and authentic signal, and the noise floor is raised. When the noise floor is raised to a certain extent, authentic signals may be submerged in the noise [8]. If the power of spoofing signal is extremely high, it is easy for the target receiver to detect an abnormality and other navigation devices are used, and effective spoofing cannot be achieved [4]. Therefore, it is of immense importance to explore the implementation of spoofing and the subsequent power control problems for receivers in the acquisition phase.

Some scholars conducted research on the necessary conditions for successful spoofing. In 2014, Ma et al. discussed the effective implementation of spoofing, which requires a 5 dB jamming-to-signal ratio to ensure that the receiver acquires the spoofing signal during the acquisition phase [9]. In 2015, Hu and others adjusted the spoofing power in real time, while realizing the traction of the receiver acquisition loop, noise floor was limited to 3 dB, and the maximum spoofing signal-to-noise ratio was limited to 22 dB, thereby achieving a continuous effective spoofing [10]. In 2016, Pang et al. categorized spoofing into acquisition phase and tracking phase, it is believed that in the acquisition phase, if receiver has not locked signal, spoofing can be successfully implemented as long as the spoofing signal power is greater than the authentic signal power [11].

Additionally, avoiding spoofing detection is also a problem that needs to be studied [12]. In 2012, Ali jafarnia jahromi believed that because spoofing signals can raise the noise floor of the receiver, the receiver can detect and identify spoofing signals more effectively by measuring the absolute power of correlation peak than by monitoring technology. In 2013, Lv and others proposed that in the signal acquisition process, if the peak of the spoofing signal and authentic signal exceeds 1.5 chips, the correlation function will have multi-peak characteristics [13]. In 2014, Daniel P. Shepard et al. used the Monte Carlo experiments to verify that the carrier frequency difference is extremely large in the process of acquiring the spoofing signal and authentic signal under the specific receiver phase locked loop parameter setting, causing the receiver to loss of lock [14]. Some scholars also studied the implementation methods of spoofing. In 2018, Sheng and others studied spoofing algorithm, through theoretical analysis, it was confirmed that for the acquisition phase, the algorithm of suppressing and spoofing needs to be adopted [15].

There are also some spoofing detection methods, such as the method of measuring the total signal energy based on spoofing signal and authentic signal proposed by Hu et al. [16]. However, when the phase difference and Doppler frequency difference between the spoofing signal and authentic signal are small, the spoofing detection performance deteriorates, and for multipath signals, the spoofing detection performance also deteriorates. Oligeri et al. used the unencrypted IRIDIUM Ring Alert (IRA) message broadcast by IRIDIUM satellite to detect spoofing [17]. Pini et al. proposed a low complexity strategy for detecting intermediate spoofing attacks based on Neyman Pearson theory [18]. Chen et al. proposed a spoofing detection method using two antennas, which can detect a single spoofing signal or spoofing signals from multiple directions. However, for dynamic scenes, the spoofing detection value is unstable [19]. Obviously, any spoofing detection technology is difficult to detect all spoofing methods, and our research focuses on the design of spoofing signal power to avoid the related power detection techniques as much as possible. Navigation security, or GNSS security, like renewable energy, has attracted more and more attention [20,21].

Currently, there is limited research involving GNSS spoofing signal power control, and there is no systematic complete power control scheme, most of which is limited to qualitative or simulation, and the actual application effect is still unclear. It is of immense importance to explore the implementation of spoofing and the subsequent power control problems for receivers in the acquisition phase.

With regard to the application of the algorithm, we need to explain that in the application of the actual spoofer, when it is difficult for the spoofer to obtain the accurate position of target receiver, it is usually used to jamming first to make the receiver lose lock and then implement spoofing. In order to maintain the concealment of spoofing, the power control of spoofing signal is a key issue at this time. Under this background, we propose a spoofing signal power control strategy under the receiver power constraint for the receiver and subsequent control in the acquisition stage. On the one hand, the algorithm can make the spoofer successfully cheat the receiver, on the other hand, the power value of the spoofing signal can be kept as hidden as possible.

A new GNSS spoofing signal power control algorithm for receiver power constraints in the acquisition phase receiver and subsequent control is proposed in this research. The designed experimental platform proves that the designed algorithm can conceal itself, take over receiver and subsequently pull positioning results with the aid of suppression interference. Furthermore, it provides a power control algorithm for suppressing post-spoofing and has a high applicability.

## 2. Effect of Spoofing Signals on Acquisition

### 2.1. Noise Floor Estimation

When the target receiver receives both spoofing signal and authentic signal, the complex signal model can be expressed as [10]:

(1) $$\begin{array}{l} r(nT_{s})=\sum_{h=J^{a}}\sqrt{P_{h}^{a}}D_{h}^{a}(nT_{s}-\tau_{h}^{a})c_{h}^{a}(nT_{s}-\tau_{h}^{a})e^{j\varphi_{h}^{a}+j2\pi f_{h}^{a}nT_{s}}+\\ \sum_{m=J^{s}}\sqrt{P_{m}^{s}}D_{m}^{s}(nT_{s}-\tau_{m}^{s})c_{m}^{s}(nT_{s}-\tau_{m}^{s})e^{j\varphi_{m}^{s}+j2\pi f_{m}^{s}nT_{s}}+\eta (nT_{s}) \end{array}$$

where subscripts *h* and *m* represent the received authentic satellite signals and spoofing signals, respectively, $J^{a}$ and $J^{s}$ are the set of authentic and spoofing signals, respectively, and the superscripts *a* and *s* represent the received authentic satellite signals and spoofing signals, respectively. $T_{s}$ is the sampling interval, *P* is the power of received signal, *c* is the pseudorandom noise (PRN) code sequence, *D* is the navigation message, φ, *f*, and τ are the carrier phase of received signal, carrier frequency Doppler shift, and code phase delay, and $\eta (nT_{s})$ is an additive white Gaussian noise with a mean of zero and a variance of $\sigma_{n}^{2}$.

The coherent integrated output value of the *l*th signal can be expressed as:

(2) $$u_{l}\left[{\tilde{f}}_{l},{\tilde{\tau }}_{l},k\right]=\frac{1}{N}\sum_{n=(k-1)N+1}^{kN}r(nT_{s})c_{l}(nT_{s}-{\tilde{\tau }}_{l})e^{-j2\pi {\tilde{f}}_{l}nT_{s}}$$

where ${\tilde{f}}_{l}$, ${\tilde{\tau }}_{l}$ represent the estimated Doppler shift and code phase delay, respectively, and *k* represents the number of integration interval related outputs.

Referring to Gaussian Sum Theory [22], the ${\hat{\sigma }}^{2}$ calculated using the averaging method can be expressed as:

(3) $${\hat{\sigma }}^{2}=\frac{1}{2}\left[\sum_{h=J^{a}}P_{h}^{a}\mathrm{var}\left[\psi_{hl}^{a}\left[{\tilde{f}}_{l},{\tilde{\tau }}_{l},k\right]\right]+\sum_{m=J^{s}}P_{m}^{s}\mathrm{var}\left[\psi_{ml}^{s}\left[{\tilde{f}}_{l},{\tilde{\tau }}_{l},k\right]\right]+\mathrm{var}\left[\bar{\eta }\left[k\right]\right]\right]$$

The noise estimator comprises three parts: ① related interference generated by authentic signal; ② related interference generated by spoofing signal; ③ related interference generated by Gaussian noise. $\psi_{hl}^{a}\left[{\tilde{f}}_{l},{\tilde{\tau }}_{l},k\right]$ and $\psi_{ml}^{s}\left[{\tilde{f}}_{l},{\tilde{\tau }}_{l},k\right]$ represent the parameters of one channel authentic and one channel spoofing signal with the local code and carrier, respectively. Because this is a complex signal, both contain *I* and *Q* branches that are orthogonal to each other, and both the *I* and *Q* branches are subject to a zero-mean Gaussian distribution. Then, $\psi_{hl}^{a}\left[{\tilde{f}}_{l},{\tilde{\tau }}_{l},k\right]$ is represented by a two-dimensional covariance matrix with the *I* and *Q* branches as random variables [23]:

(4) $$\left\{\begin{array}{l} \psi_{hl}^{a}\left[{\tilde{f}}_{l},{\tilde{\tau }}_{l},k\right]∼N\left(\left[\begin{array}{l} 0\\ 0 \end{array}\right],\left[\begin{array}{cc} {\bar{\sigma }}_{\psi ,normalization}^{2} & 0\\ 0 & {\bar{\sigma }}_{\psi ,normalization}^{2} \end{array}\right]\right)\\ {\bar{\sigma }}_{\psi ,normalization}^{2}={\bar{\sigma }}_{\psi }^{2}/\max\left({\bar{\sigma }}_{\psi ,normalization}^{2}\right){\bar{\sigma }}_{\psi }^{2} \end{array}\right.$$

The cross-correlation variance ${\bar{\sigma }}_{\psi ,normalization}^{2}$ of the normalized spreading codes of the *I* and *Q* branches is statistically averaged as 0.00033. $\psi_{ml}^{s}\left[{\tilde{f}}_{l},{\tilde{\tau }}_{l},k\right]$ obeys the same distribution.

The correlation value generated by noise is still a zero-mean Gaussian noise, and $\bar{\eta }[k]$ can be expressed by the same distribution as

(5) $$\bar{\eta }[k]∼N\left(\left[\begin{array}{l} 0\\ 0 \end{array}\right],\frac{\sigma_{n}^{2}}{N}\left[\begin{array}{cc} 1 & 0\\ 0 & 1 \end{array}\right]\right)$$

Then, the noise variance estimate can be expressed as [10]:

(6) $$\left\{\begin{array}{l} {\hat{\sigma }}^{2}=\frac{N_{0}}{2NT_{s}}+\left(\sum_{h=J^{a}}P_{h}^{a}+\sum_{m=J^{s}}P_{m}^{s}\right){\bar{\sigma }}_{\psi ,normalization}^{2}\\ {\bar{\sigma }}_{\psi ,normalization}^{2}={\bar{\sigma }}_{\psi }^{2}/\max\left({\bar{\sigma }}_{\psi ,normalization}^{2}\right){\bar{\sigma }}_{\psi }^{2} \end{array}\right.$$

Therefore, the noise variance estimation value is related to the ambient noise power $N_{0}$, coherent integration time $T_{c}=NT_{s}$, variance ${\bar{\sigma }}_{\psi ,normalization}^{2}$ of the *I/Q* branch normalized spreading code, total power of each spoofing signal $\sum_{m=J^{s}}P_{m}^{s}$, and total power $\sum_{h=J^{a}}P_{h}^{a}$ of each channel. In Equation (12), we normalized ${\hat{\sigma }}^{2}$ to obtain the estimated noise variance. We add a flow chart for the acquisition and tracking loop structure used in this research to make the derivation in Section 2.1 and Section 2.2 more clear.

**Figure 1:**
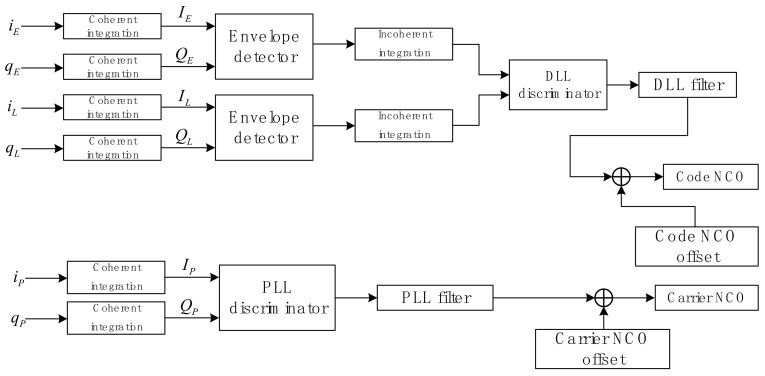
Flow chart for the acquisition and tracking loop structure.

As shown in Figure 1, the correlation results $i_{E}$, $i_{P}$, $i_{L}$, $q_{E}$, $q_{P}$ and $q_{L}$ are coherent integrated to output coherent integration values $I_{E}$, $I_{P}$, $I_{L}$, $Q_{E}$, $Q_{P}$ and $Q_{L}$. Then, after envelope detection and incoherent integration, the coherent integral values $I_{P}$ and $Q_{P}$ on the prompt branch are used as inputs to the PLL discriminator, and the coherent integral values of the other two related branches are used as inputs to the DLL discriminator. Finally, the DLL and the PLL filter the respective discrimination results, and adjust the output of code NCO and carrier NCO according to the filtering results, so that the locally output carrier and code are consistent with the received signal.

**Figure 2:**
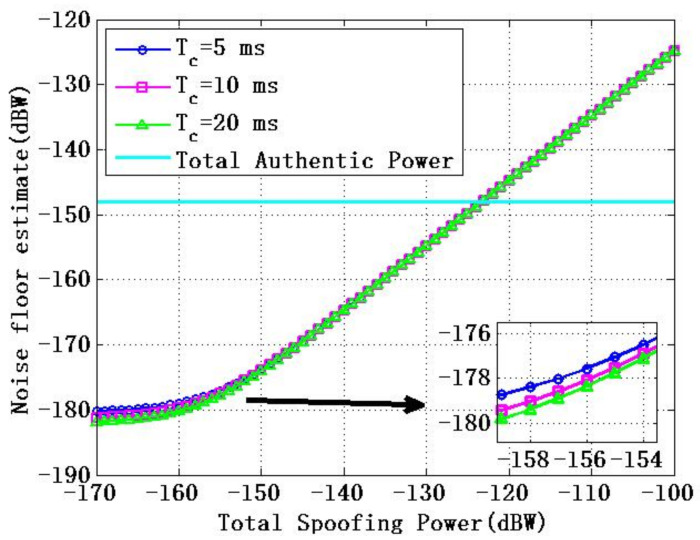
Relationship between total power of spoofing signal and estimation of receiver noise floor.

Figure 2 shows the relationship between the total power of spoofing signal and estimation of the noise floor of receiver when $T_{c}=5\;\mathrm{ms}$, $T_{c}=10\;\mathrm{ms}$, $T_{c}=20\;\mathrm{ms}$ are used. Figure 2 shows that with the increase of the total power of the spoofing signal, noise floor estimation also increases and gradually exceeds total power of authentic signal.

### 2.2. Acquisition Performance Analysis

Receiver’s acquisition search for a Global Navigation Satellite System (GNSS) signal is a signal search performed in a two-dimensional space consisting of frequency and time. 

According to the signal detection theory [24], in the presence and absence of satellite signals, the non-coherent integral amplitude *V* is $\chi^{2}$ distribution and Rayleigh distribution, respectively. When a satellite signal exists and the number of non-coherent integrations $N_{nc}$ is 1, the number of non-coherent integralion means that the receiving channel generates a pair of coherent integration results every other coherent integration time, and the number of incoherent integration results in each search unit is the number of incoherent integrals. The detection amount *V* of the satellite signal obeys the non-central $\chi^{2}$ distribution, and the detection probability $P_{d}$ is:

(7) $$P_{d}(V)=\int_{D_{t}}^{\infty }\frac{V}{\sigma_{n}^{2}}e^{-\frac{V^{2}+P_{l}}{2\sigma_{n}^{2}}}I_{0}\left(\frac{VP_{l}}{\sigma_{n}^{2}}\right)dV\;,\;(V>0)$$

Here, $I_{0}\left(\cdot \right)$ is a zero-order first-order modified Bessel function, $2\sigma_{n}^{2}$ is the noise power, and $P_{l}$ is the power after the coherent integration of the lth signal. $D_{t}$ is the detection threshold, and the above formula can be expressed as follows by pre check signal-to-noise *SNR* (where *SNR* is the dimensionless ratio) [25]:

(8) $$P_{d}=\int_{D_{t}}^{\infty }\frac{V}{\sigma_{n}^{2}}e^{-\left(\frac{V^{2}}{2\sigma_{n}^{2}}+SNR\right)}I_{0}\left(\frac{V\sqrt{2\cdot SNR}}{\sigma_{n}}\right)dV\;,\;(V>0)$$

In fact, Equations (7) and (8) are equivalent, but the calculation methods are different. When satellite signal does not exist, the detection metric *V* presents Rayleigh distribution, and the false alarm rate $P_{fa}$ corresponding to the threshold $V_{t}$ is expressed as:

(9) $$P_{fa}=\int_{V_{t}}^{\infty }\frac{v}{\sigma_{n}^{2}}e^{\left(-\frac{v^{2}}{2\sigma_{n}^{2}}\right)}dv$$

For multi-channel spoofing signals and authentic signals, in order to detect correctly, all detection units should not appear as afalse alarm. Therefore, considering that detection units are independent of each other, the total false alarm probability $P_{fa-total}$ is defined as:

(10) $$P_{fa-total}=1-{\left(1-P_{fa}\right)}^{N_{c}}$$

$N_{c}$ is the number of search units contained in the two-dimensional search range. The number of search units means that the area surrounded by the frequency indeterminate interval and the code phase indeterminate interval constitutes a two-dimensional search range for a received signal. The intersection of each code band and each frequency band is called a search unit. The number of search units means the number of search units within the above search range. Then the detection threshold $V_{t}$ is calculated from the total false alarm rate $P_{fa-total}$ of signal acquisition as follows [25]:

(11) $$V_{t}^{2}=-2\sigma^{2}\ln\left[1-{\left(1-P_{fa-total}\right)}^{\frac{1}{N_{c}}}\right]$$

The total power of spoofing signal and authentic signal are defined as $TSP=10\mathrm{lg}(\sum_{m=J^{s}}P_{m}^{s})$ and $TAP=10\mathrm{lg}(\sum_{h=J^{a}}P_{h}^{a})$, respectively. The signal-to-noise ratio of power $P_{m}^{s}$ and $P_{h}^{a}$ of each spoofing signal and noise variance estimation ${\hat{\sigma }}^{2}$ are expressed as $SNR_{i}^{s}$ and $SNR_{i}^{a}$, respectively [10]:

(12) $$SNR_{i}^{s}=\frac{P_{m}^{s}}{2{\hat{\sigma }}^{2}}\;,\;SNR_{i}^{a}=\frac{P_{h}^{a}}{2{\hat{\sigma }}^{2}}$$

In the above formula, let $\sigma_{n}=1$ (normalized). The conditional probability that GNSS signal is detected by target receiver and signal is a spoofing signal is used as the basis for spoofing signal acquisition performance [26], so that authentic signal and spoofing signal detection probabilities of the *i* th channel are $P_{d,i}^{a}$ and $P_{d,i}^{s}$, respectively. This conditional probability is called the relative acquisition probability $P_{i}$ of spoofing signal:

(13) $$P_{i}=\frac{P_{d,i}^{s}}{P_{d,i}^{s}+P_{d,i}^{a}}$$

**Figure 3:**
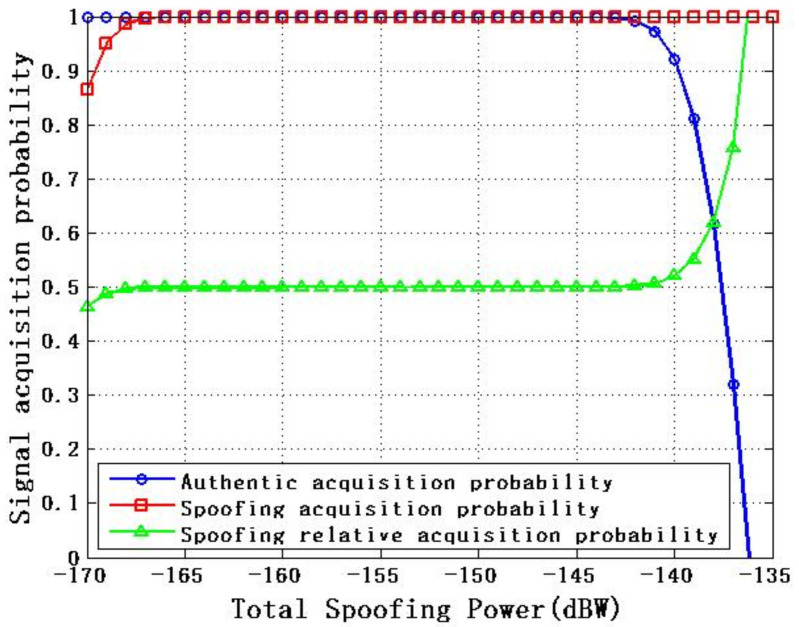
Relationship between total power of spoofing signal and acquisition probability.

Each branch spoofing signal satisfies the high acquisition performance while being constrained by noise floor and relative acquisition probability. Figure 3 shows the relationship between total power of spoofing signal and the probability of signal acquisition.

Figure 3 shows that when total power of spoofing signal increases from −170 dBW to –159 dBW, the acquisition probability of the spoofing signal increases from 0.86 to 1, the relative acquisition probability of the spoofing signal increases from 0.46 to 0.5, and the authentic signal acquisition probability is always 1. When total power of spoofing signal increases from −159 dBW to −143 dBW, the acquisition probability of spoofing signal, relative acquisition probability of the spoofing signal and acquisition probability of the authentic signal remain unchanged. When total power of spoofing signal increases from −143 dBW to −135 dBW, acquisition probability of the spoofing signal is always 1, while the acquisition probability of the authentic signal decreases from 1 to 0, and relative acquisition probability of spoofing signal increases from 0.5 to 1.

When the total power of spoofing signal is −170 dBW, because authentic signal is the default value −158 dBW, the acquisition probability of spoofing signal can be calculated as 0.86 according to Equation (7). However, it should be noted that this does not mean that the receiver is easier to acquire spoofing signal, because the acquisition probability of authentic signal is 1 and the relative acquisition probability of the spoofing signal is about 0.44. Therefore, we believe that the receiver still preferentially captures authentic signal, that is to say, it still depends on the relative acquisition probability of the spoofing signal. In Figure 3, because authentic signal is the default value −158 dBW, when the ‘Total Spoofing Power’ is −175 dbW, the power of the authentic signal is −158 dBW.

### 2.3. Signal Power Transmission Loss Model

According to the free-space propagation theory of satellite signals [27], if the transmit power of the spoofing signal transmitted by the spoofing device is $P_{T}$ (unit: dBW), the gain of the transmitting antenna in a certain direction is $G_{T}$, and the corresponding gain of the target receiver of the receiving antenna at R is $G_{R}$, λ is the signal wavelength, *d* is the spatial distance between the spoofer and receiver, and the power of spoofing signal received by receiving antenna is $P_{R}$. The link power budget equation expressed in decibels is:

(14) $$P_{R}=P_{T}+G_{T}+G_{R}+20\mathrm{lg}\left(\frac{\lambda }{4\pi d}\right)$$

where $20\mathrm{lg}\left(\frac{\lambda }{4\pi d}\right)$ is the free space propagation loss, and signal received power, $P_{R}$ reflects the absolute strength of signal.

In the process of the spoofing, the power needs to be adjusted in real time according to the relative position change of the spoofer and target receiver, such that the signal power received by receiver is maintained within a certain range.

### 2.4. Relationship between Satellite Elevation Angle and Received Signal Power

The satellite signal strength received by receiver has a significant relationship with satellite elevation angle [27]. Therefore, for the spoofer that transmits the multi-channel spoofing signal, it is necessary to consider the influence of the satellite elevation angle on the power distribution of the spoofing signal. For some intermediate spoofing devices that receive authentic satellite signals to reconstruct spoofing signal, the elevation angle information of the simulated authentic satellite signals must be obtained according to the ephemeris received in real time, and the generated spoofing signals must consider the satellite elevation angle inside. The relationship between the satellite elevation angle and the power of spoofing signal applicable to this research is discussed below.

**Figure 4:**
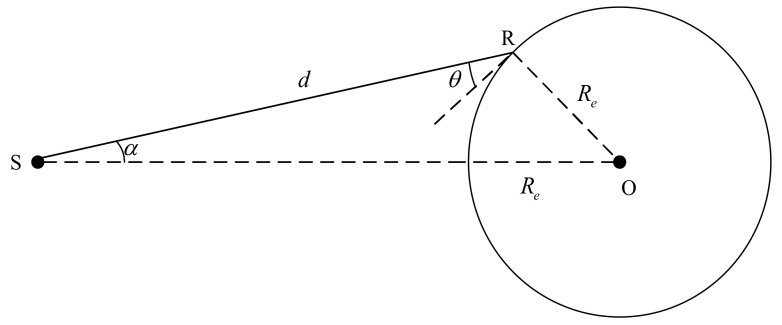
Free space propagation of satellite signals.

As illustrated in Figure 4, the ground receiver is located at R, the satellite is located at S, the spatial distance between the satellite and receiver is *d*, the Earth’s center is O, the Earth’s radius is $R_{e}$, α is the angle between SR and SO, and θ is the angle between SR and RO. By applying the sine theorem to the triangle ORS in above figure, we obtain:

(15) $$\frac{R_{e}}{\sin\alpha }=\frac{d}{\sin(180^{{}^{\circ}}-\alpha -\theta -90^{{}^{\circ}})}$$

Then, the signal propagation distance *d* can be expressed as:

(16) $$d=\frac{R_{e}\cos(\alpha +\theta )}{\sin\alpha }$$

As the free space propagation loss obtained by Equation (14) is $20\mathrm{lg}\left(\frac{\lambda }{4\pi d}\right)$, the relationship between the satellite elevation angle and received signal power of the receiver is:

(17) $$P_{R}=P_{T}+G_{T}+G_{R}+20\mathrm{lg}\left(\frac{\lambda \sin\alpha }{4\pi R_{e}\cos(\alpha +\theta )}\right)$$

For the subsequent analysis, the above formula can be simplified as:

(18) $$P_{R}\approx k_{1}+\frac{k_{2}}{\cos(\alpha +\theta )}$$

where $k_{1}$ and $k_{2}$ represent coefficients. Therefore, it can be considered that the lower the satellite elevation angle, the smaller the corresponding satellite signal power received by receiver, and the specific quantization expression is defined in Equation (17).

It is shown in Section 2.1 and Section 2.2 that for the multi-channel spoofing signal model, if the power of spoofing signal is increased without restriction, the noise floor of the receiver will increase, which may be alerted by the power or noise floor monitoring function of some commercial receivers and may also reduce the probability of spoofing signal acquisition and interference efficiency. Therefore, the following gives the optimization algorithm of power allocation of each spoofing signal.

## 3. GNSS Spoofing Signal Power Control Algorithm

### 3.1. Constraint Analysis

In a spoofing scenario, the variance ${\bar{\sigma }}_{\psi }^{2}$ of the general ambient noise power $N_{0}$, coherent integration time $T_{c}=NT_{s}$, and *I/Q* branch normalized spreading code remains unchanged. For spoofer, total power $\sum_{h=J^{a}}P_{h}^{a}$ of each channel is uncontrollable. Only spoofing signal $P_{m}^{s}$ and total power $\sum_{m=J^{s}}P_{m}^{s}$ are designed to satisfy the following basic conditions: ① The maximum signal-to-noise ratio $SNR_{max}^{s}$ of each branch spoofing signal is lower than the receiver signal-to-noise ratio detection threshold $SNR_{thres}$ (or the maximum signal-to-noise ratio $SNR_{max}^{a}$ of authentic satellite signal); ② the minimum signal-to-noise ratio $SNR_{min}^{s}$ of each branch spoofing signal is higher than the average signal-to-noise ratio $SNR_{ave}^{a}$ of the authentic satellite signal; ③ the power of the spoofing signal of each branch corresponding satellite elevation angle matching; ④ if the target receiver is dynamic, spoofing signal power should be adjusted according to the signal power transmission loss model; ⑤ the relative acquisition probability of each spoofing signal satisfies $P_{i}>0.5$. When the single-channel spoofing signal is relatively close to the probability of acquisition $P_{i}>0.5$, the ability of the spoofing signal to be preferentially acquired is explained. The 5th constraint here means that we usually think that when the single-channel spoofing signal is relatively close to the probability of acquisition $P_{i}>0.5$, it indicates that for the receiver, the acquisition probability of spoofing signal is greater than the acquisition probability of authentic signal, so the spoofing signal will be acquired preferentially, which is also a condition for successful spoofing.

The above constraint formula is expressed as:

(19) $$\left\{\begin{array}{l} SNR_{max}^{s}<SNR_{thres}\\ SNR_{min}^{s}>SNR_{ave}^{a}\\ P_{m}^{s}\approx k_{1}+\frac{k_{2}}{\cos(\alpha +\theta )}\\ P_{m}^{s}=P_{T}+G_{T}+G_{R}+20\mathrm{lg}\left(\lambda /(4\pi d)\right)\\ P_{i}>0.5 \end{array}\right.$$

### 3.2. Building the Objective Function

Under the premise of satisfying the above constraints, the following three points should be optimized: ① the noise variance estimation ${\hat{\sigma }}^{2}$ should be minimum; ② the relative acquisition probability of each branch spoofing signal should be optimal; ③ the overall acquisition performance of the multi-channel spoofing signal achieved should be excellent. The overall acquisition performance of the multi-channel signal is represented by the sum of the relative acquisition performance of each spoofing signal: $\sum_{i=1}^{n}P_{i}$. Therefore, the objective function can be defined as:

(20) $$\left\{\begin{array}{l} F_{{\hat{\sigma }}^{2}}=\frac{N_{0}}{2NT_{s}}+\left(\sum_{h=J^{a}}P_{h}^{a}+\sum_{m=J^{s}}P_{m}^{s}\right){\bar{\sigma }}_{\psi }^{2}\\ F_{P_{t}}=\frac{P_{d,i}^{s}}{P_{d,i}^{s}+P_{d,i}^{a}}\\ F_{P_{total}}=\sum_{i=1}^{n}P_{i} \end{array}\right.$$

where $F_{{\hat{\sigma }}^{2}}$, $F_{P_{t}}$, and $F_{total}$ represent three objective functions, respectively, to minimize $F_{{\hat{\sigma }}^{2}}$, $F_{P_{t}}$ and $F_{total}$ reach the maximum.

### 3.3. Power Allocation Optimization Algorithm

The multi-objective optimization algorithm of sequential quadratic programming (SQP) is used to determine the power of each spoofing signal. SQP algorithm is an algorithm that converts the nonlinear constrained optimization problem into a relatively simple quadratic programming problem, and the quadratic programming problem is an optimization problem in which the objective function is a quadratic function, and the constraint function is a linear function. The specific process of the power allocation optimization algorithm based on SQP is as follows: $F_{{\hat{\sigma }}^{2}}$, $F_{P_{t}}$, $F_{total}$ in the above formula are taken as the objective function, and the expected values of the objective function are $F_{{\hat{\sigma }}^{2}}=0$, $F_{P_{t}}=-1$, $F_{total}=-N$, respectively, N indicating the number of spoofing signals. The weight of each expected value is set as: $W_{F_{{\hat{\sigma }}^{2}}}=0$, $W_{F_{P_{t}}}=1$, $W_{F_{total}}=N$. Under the constraint condition in Equation (19), the smaller the values of $F_{{\hat{\sigma }}^{2}}$, $F_{P_{t}}$, $F_{total}$, the better spoofing signal power allocation scheme is proved.

## 4. Experimental Verification

**Figure 5:**
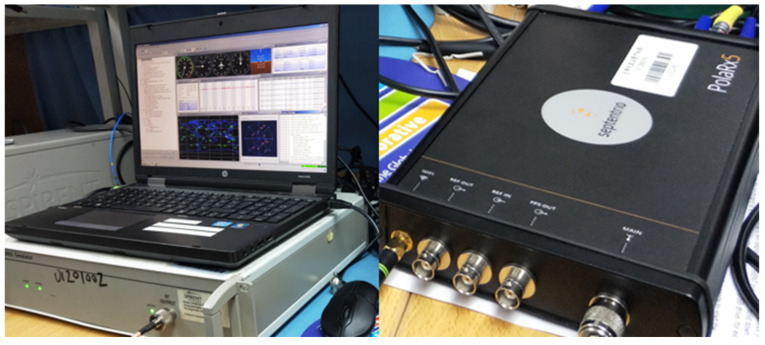
Experimental platform setup.

The experimental platform is setup as depicted in Figure 5. It comprises GNSS signal simulator, host computer control software, test receiver, and connection feeder. GNSS signal simulator is used as an authentic signal and spoofing signal generating device. The host computer control software controls the code offset (unit: m), carrier phase offset (unit: m), and code rate (unit: m/s) of each spoofing signal relative to the authentic signal and carrier phase rate (unit: m/s), and relative power gain (unit: dB), power increase/decrease rate (unit: dB/s) by writing instructions. In addition, the Space Vehicle Identification (SVID), number of satellites, and signal power value of authentic signal and spoofing signal can be selected initially. The experiment uses the PolaRx5 receiver of Septentrio as the target receiver, which has advanced interference monitoring and anti-interference ability.

The following two groups of experiments are designed to compare the spoofing effect of the newly designed GNSS spoofing signal power control algorithm and spoofing signal high power control algorithm on a test receiver. In experiment 1, the high-power control algorithm was performed for the spoofing signal, and in experiment 2, the new power control algorithm was performed for the spoofing signal. In the two groups of experiments, except the GNSS spoofing signal power control algorithm, the other experimental conditions were the same.

### 4.1. Experiment on High Power Control Algorithm of Spoofing Signal

**Figure 6:**
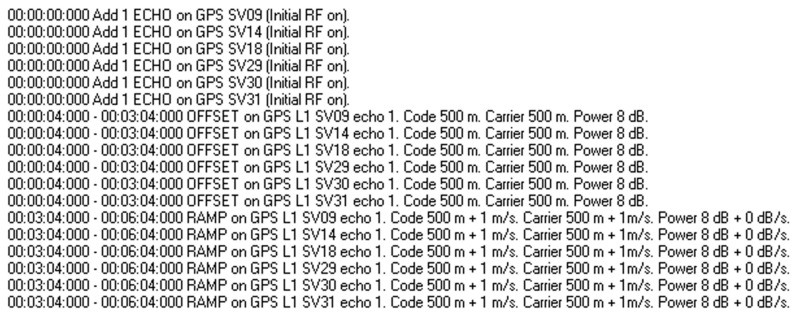
Experimental instruction design in experiment 1.

In experiment 1, high power control algorithm was used for spoofing signal. The design experimental procedure is as follows: ① First, PolaRx5 receiver is cold-started to ensure that the receiver is in the state of signal loss-locking after the receiver is suppressed for a long duration by the signal; ② after cold start, signal simulator is used to generate 6 channels of L1 authentic signals and 6 channels of same SVID spoofing signals, each branch spoofing signal has an initial code phase offset of 500 m and an initial carrier phase offset of 500 m with respect to the authentic signal, the spoofing signal is consistent with code rate and carrier phase rate of the authentic signal, compared with authentic signal, the power advantage of spoofing signal is 8 dB, all signals are simultaneously injected into receiver from the connected feeder to simulate the spoofing of the receiver in the signal acquisition phase. The process lasts for 3 min; ③ after 3 min, the other parameters of each spoofing signal are kept unchanged, only the code rate is adjusted to 1 m/s and the carrier phase rate is adjusted to 1 m/s, which increases code phase and carrier phase by a fixed slope. The duration of this process is 3 min. After 246 s, the experimental key instruction design is depicted in Figure 6.

In Figure 6, “OFFSET” indicates the code phase offset, carrier phase offset, relative power gain relative to authentic signal, command start time, and duration time; “RAMP” indicates the initial code phase offset, initial carrier phase offset, initial relative power gain, code rate and carrier phase rate, power increase/decrease rate, relative to authentic signal, and command start time and duration time.

The following analyzes the changes of Doppler frequency, carrier-to-noise ratio, and Earth-Centered, Earth-Fixed (ECEF) of the receiver in the test process.

The ground truth in ECEF has been set, the reference position in ECEF is −1,445,000 m in the *X*-axis direction, 6,150,000 m in the *Y*-axis direction, 180,000 m in the *Z*-axis direction. The ECEF coordinate given in this experiment is the positioning error vector subject to the reference position.

The statistical results of Doppler frequency range (difference between maximum and minimum), maximum $C/N_{0}$, minimum $C/N_{0}$ and average $C/N_{0}$ of six signals received by test receiver are shown in Table 1.

**Figure 7:**
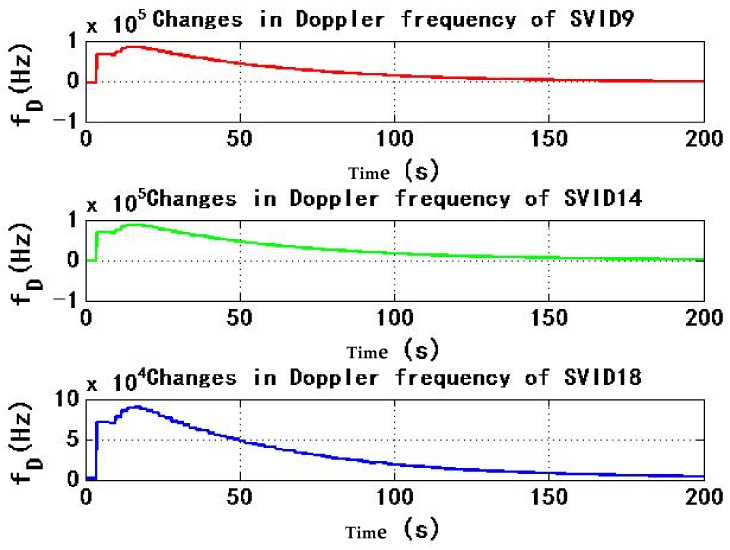
Changes in Doppler frequency of SVID9, 14, 18 in experiment 1.

**Figure 8:**
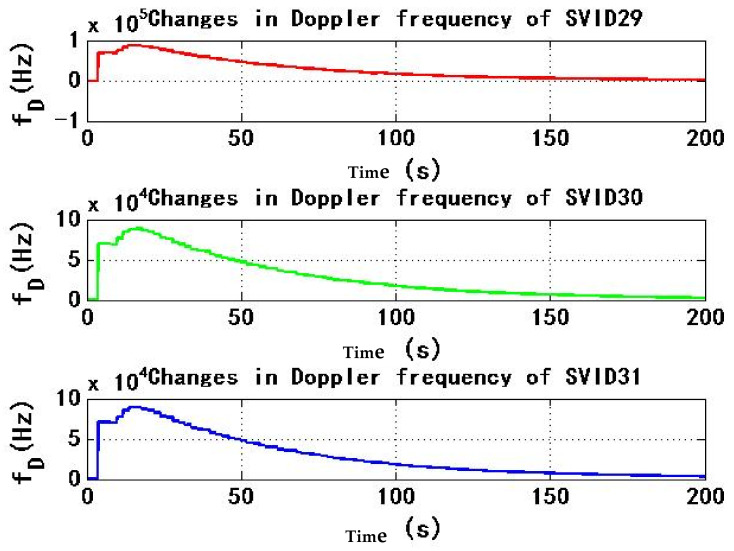
Changes in Doppler frequency of SVID29, 30, 31 in experiment 1.

Figure 7 and Figure 8 show the change of Doppler frequency with time of the six signals received by test receiver. During the period of 4–184 s, spoofing signal and authentic signal affect receiver at the same time, and Doppler frequency of six signals fluctuates. According to the statistics during 0–200 s, the range of Doppler frequency is 87,900 Hz. During 184–200 s, code rate and carrier phase rate of spoofing signals change, and Doppler frequency of signals remains stable.

**Figure 9:**
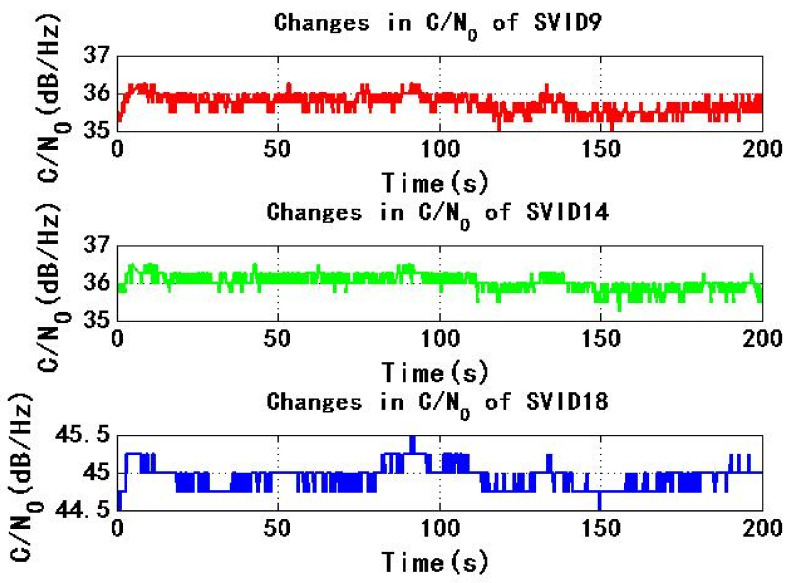
Changes in ${C/N}_{0}$ of SVID9, 14, 18 in experiment 1.

**Figure 10:**
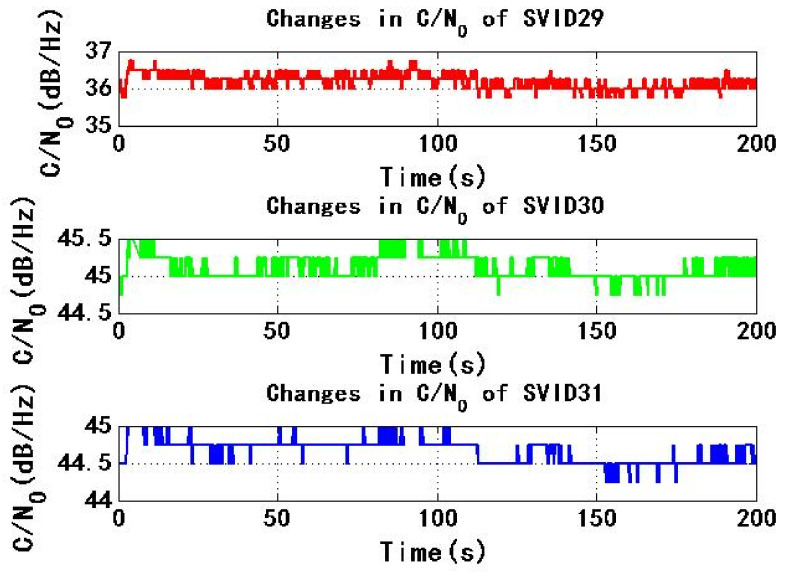
Changes in ${C/N}_{0}$ of SVID29, 30, 31 in experiment 1.

Figure 9 and Figure 10 show the change of $C/N_{0}$ of the six signals received by test receiver with time. During the period of 0–200 s, $C/N_{0}$ changes smoothly. The maximum $C/N_{0}$ of SVID18, SVID30 and SVID31 signals are not less than the threshold $C/N_{0}$ of the receiver for 45 dB/Hz, then the received signal $C/N_{0}$ is not less than the threshold $C/N_{0}$ of the receiver, this will make it easy for the power monitoring technology of the receiver to detect the spoofing signal.

**Figure 11:**
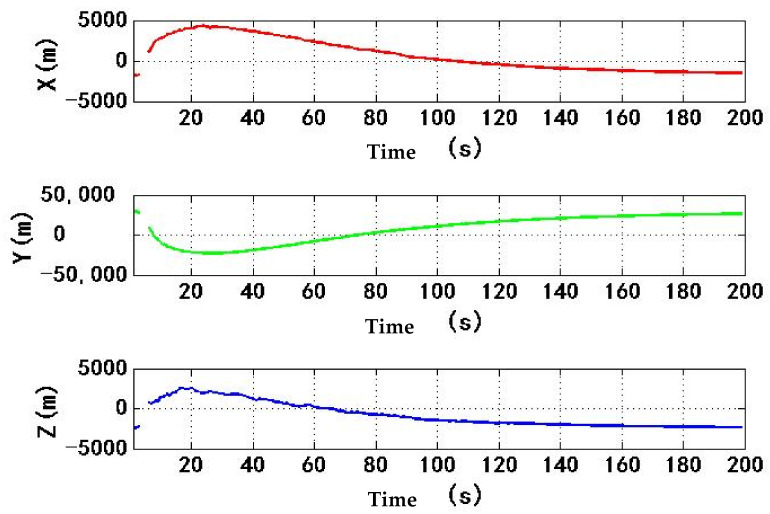
Changes in ECEF three dimensional coordinates of receiver in experiment 1.

Figure 11 shows the change of ECEF coordinate positioning results with time. During the period of 4–184 s, the spoofing signal and authentic signal affect the receiver at the same time. The range of ECEF three-dimensional coordinates is 12,260 m, 104,200 m and 10,180 m, respectively. The high-power spoofing signal causes a large fluctuation in the positioning results of receiver. During the period of 184–200 s, the corresponding spoofing signal begins to pull the positioning result stage, and the positioning result of the receiver is gradually pulled. The range of three-dimensional coordinates of ECEF is 213.5 m, 1848 m and 93.45 m, respectively, so the positioning result of the receiver has changed greatly.

In the experiment, we have explained in the designed experimental steps. In order to verify that the receiver in the state of lost lock recapture and cold start is spoofed, after the cold start of receiver, spoofing signal and authentic signal are injected into receiver at the same time. Because the code rate and carrier phase rate of spoofing signal are changed, if the receiver is controlled by spoofing signal, The positioning result of receiver will change. Because the positioning result of the receiver is constantly biased in the experiment, we can judge that the receiver is controlled by spoofing signal, but the high-power spoofing signal is not hidden enough.

### 4.2. Experiment of New GNSS Spoofing Signal Power Control Algorithm

In experiment 2, a new power control algorithm is performed for the spoofing signal. The design experimental procedure is as follows: ① First, the PolaRx5 receiver is cold-started to ensure that receiver is in the state of signal loss-locking after the receiver is suppressed for a long duration by the signal; ② after a cold start, a signal simulator is used to generate 6 channels of L1 authentic signals and 6 channels of same SVID spoofing signals, each branch spoofing signal has an initial code phase offset of 500 m and an initial carrier phase offset of 500 m with respect to the authentic signal, the spoofing signal is consistent with the code rate and carrier phase rate of the authentic signal, the power of spoofing signal relative to the authentic signal is set according to the power allocation optimization algorithm, all signals are simultaneously injected into the receiver from the connected feeder to simulate the spoofing of receiver in the signal acquisition phase. The process lasts for 3 min; ③ after 3 min, the other parameters of each spoofing signal are kept unchanged, only the code rate is adjusted to 1 m/s and the carrier phase rate is adjusted to 1 m/s, which increases code phase and carrier phase by a fixed slope. The duration of this process is 3 min. After 246 s, the experimental key instruction design is depicted in the figure below.

**Figure 12:**
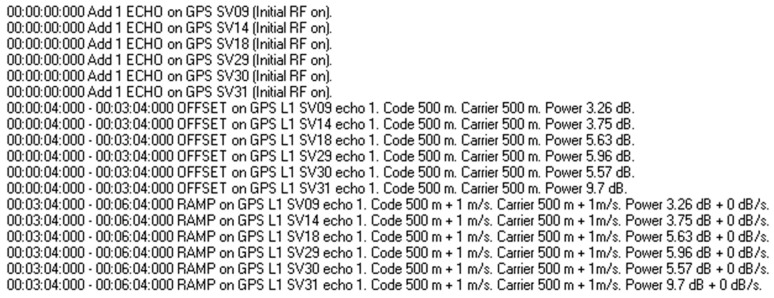
Experimental instruction design in experiment 2.

In Figure 12, “OFFSET” indicates the code phase offset, carrier phase offset, relative power gain relative to authentic signal, command start time, and duration time; “RAMP” indicates the initial code phase offset, initial carrier phase offset, initial relative power gain, code rate and carrier phase rate, power increase/decrease rate, relative to authentic signal, and command start time and duration time.

The following analyzes the changes of Doppler frequency, carrier-to-noise ratio, and ECEF of the receiver in the test process.

The ground truth in ECEF has been set, the reference position in ECEF is −1,448,700 m in the *X*-axis direction, 6,209,100 m in the *Y*-axis direction, 175,000 m in the *Z*-axis direction. The ECEF coordinate given in this experiment is the positioning error vector subject to the reference position.

The statistical results of Doppler frequency range (difference between maximum and minimum), maximum $C/N_{0}$, minimum $C/N_{0}$ and average $C/N_{0}$ of six signals received by test receiver are shown in Table 2.

**Figure 13:**
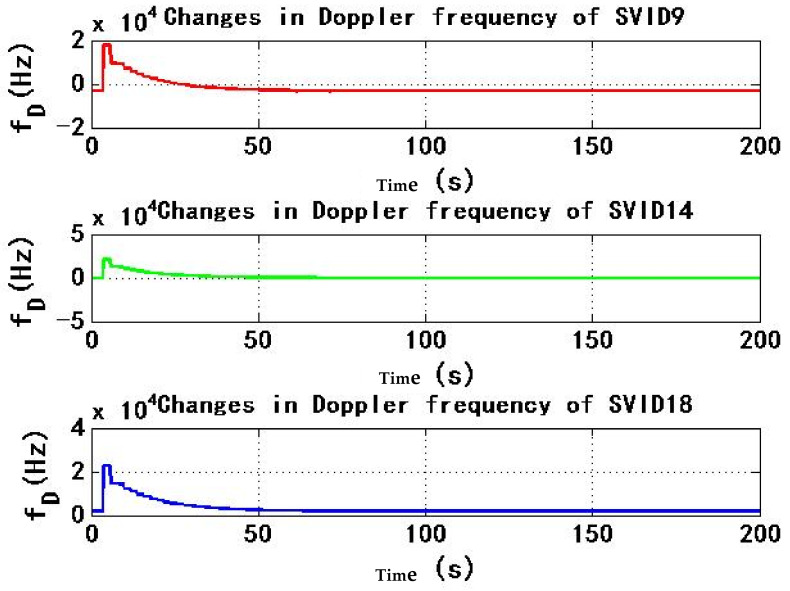
Changes in Doppler frequency of SVID9, 14, 18 in experiment 2.

**Figure 14:**
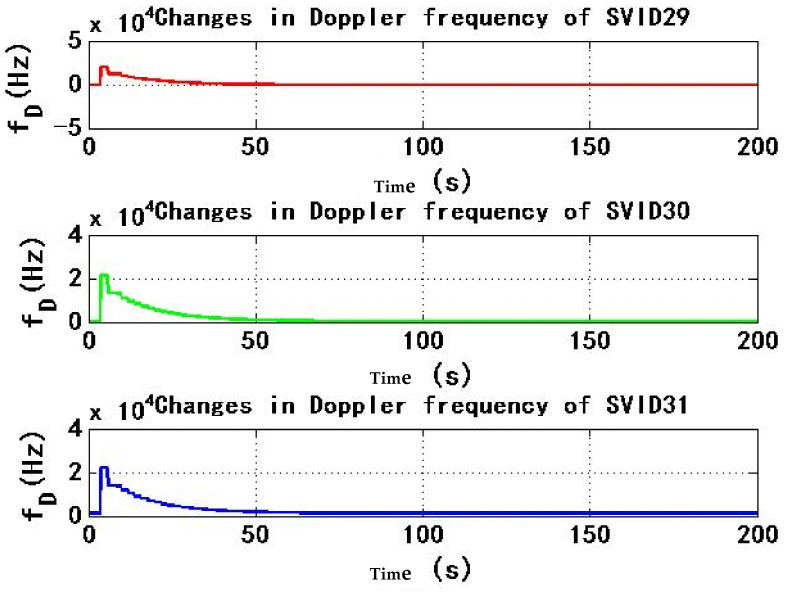
Changes in Doppler frequency of SVID29, 30, 31 in experiment 2.

Figure 13 and Figure 14 show the change of Doppler frequency with time of six signals received by the test receiver. During the period of 4–184 s, spoofing signal and authentic signal affect receiver at the same time, and Doppler frequency of six signals fluctuates. Compared with experiment 1, Doppler frequency fluctuation period is only 0–50 s, and the duration is shortened by 130 s, with a reduction percentage of 72.2%, the average value of Doppler frequency range is 21,183 Hz from 0 s to 200 s. Compared with experiment 1, Doppler frequency range is reduced by 66,717 Hz, with a reduction percentage of 75.9%; During 184–200 s, code rate and carrier phase rate of spoofing signals change, and Doppler frequency of the signal remains stable.

**Figure 15:**
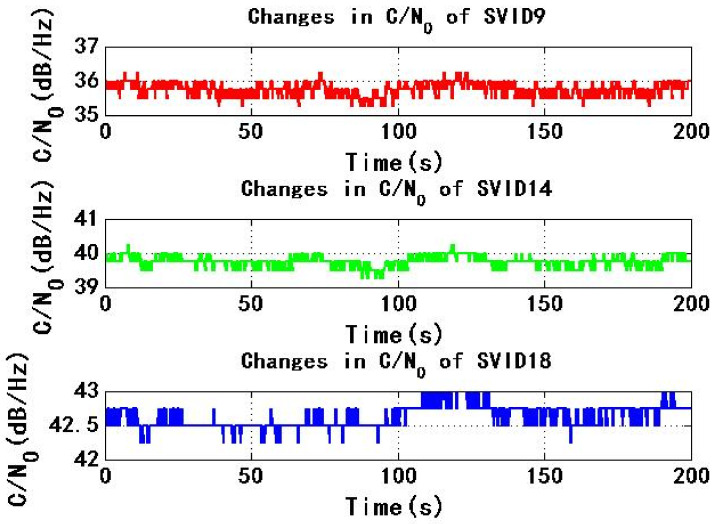
Changes in ${C/N}_{0}$ of SVID9, 14, 18 in experiment 2.

**Figure 16:**
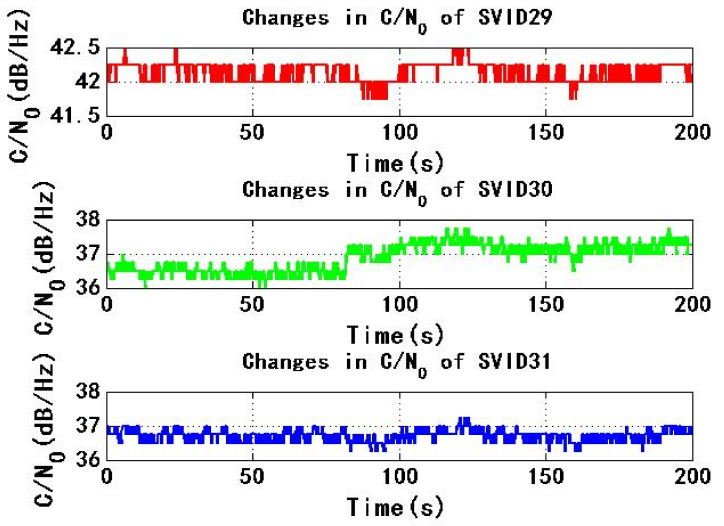
Changes in ${C/N}_{0}$ of SVID29, 30, 31 in experiment 2.

Figure 15 and Figure 16 show the change of $C/N_{0}$ of six signals received by test receiver with time. During the period from 0 s to 200 s, $C/N_{0}$ changes smoothly. If the maximum $C/N_{0}$ of six signals is less than the threshold $C/N_{0}$ of receiver for 45 dB/Hz, received signal $C/N_{0}$ is less than the threshold $C/N_{0}$ of receiver, so receiver is not easy to detect spoofing signal.

**Figure 17:**
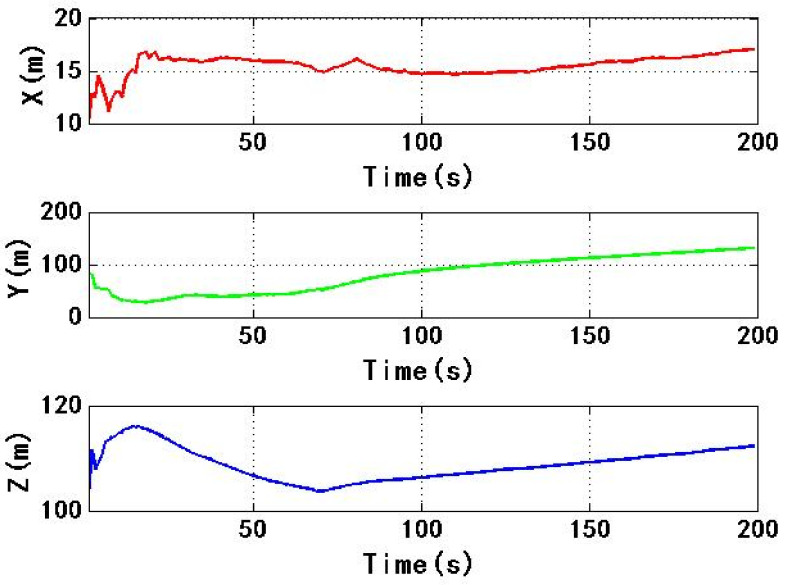
Changes in ECEF three dimensional coordinates of receiver in experiment 2.

Figure 17 shows the change of ECEF coordinate positioning results with time. During the period from 4 s to 184 s, the spoofing signal and authentic signal affect the receiver at the same time. The range of ECEF three-dimensional coordinates is 13.64 m, 194.7 m and 33.81 m, respectively. Compared with experiment 1, the change range of positioning results caused by spoofing signal is obviously small, which indicates that the process of spoofing signal taking over receiver is more stable, this is helpful to improve the concealment of spoofing. The time is between 184 s and 200 s. At this time, the corresponding spoofing signal begins to be biased. The positioning result of the receiver is gradually biased. The three-dimensional coordinate range of ECEF is 1.498 m, 12.68 m and 2.747 m, respectively. Because the code phase and carrier phase of six spoofing signals change 16 m during this period, the three-dimensional coordinate range of ECEF in experiment 2 is more reasonable than that in experiment 1. It shows that receiver is taken over by spoofing signal, and spoofing is implemented successfully.

Based on the analysis of experiment 1 and experiment 2, compared with the conventional spoofing signal high power control algorithm, the new GNSS spoofing signal power control algorithm can shorten Doppler frequency fluctuation time by 72.2%, reduce the range by 75.9%, and reduce $C/N_{0}$ of the received signal. When spoofing signal takes over the receiver, the range of the three-dimensional coordinates of ECEF is significantly reduced, which indicates that the newly designed GNSS spoofing signal power control algorithm can make the spoofing behavior more hidden and make it more difficult for the target receiver to detect the spoofing behavior.

## 5. Conclusions

In the actual application scenario of spoofing, it is difficult for the spoofer to obtain the accurate position of the antenna phase center of target receiver due to the limitation of observation conditions. Generally, the method of suppressing interference first to make the receiver lose lock and then performing spoofing is widely adopted. Under this application background, in this paper, a spoofing signal power control strategy is proposed for the receiver in the acquisition phase and subsequent control under the receiver power constraint is proposed. The actual practical experiments show that, compared with the conventional spoofing signal high power control strategy, when the newly designed spoofing signal power control strategy is adopted, the Doppler frequency fluctuation duration of the received signal is shortened by 72.2%, and the range is reduced by 75.9%, the $C/N_{0}$ of the received signal is less than the threshold value of $C/N_{0}$. During the process of spoofing signal taking over the receiver, ECEF is significantly reduced, which indicates that the newly designed spoofing signal power control strategy can make the spoofing behavior more concealed and will make it more difficult for the target receiver to detect the spoofing behavior. The designed power control strategy can covertly take over the receiver with the assistance of suppressing interference and then pull off the positioning results. It has good feasibility and effectiveness. After accurately mastering the receiver parameters, the more covert spoofing of the receiver can be realized by changing the constraint amount.

## Figures and Tables

**Table 1:** Statistical table of experiment 1 Results.

Experiment 1	SVID9	SVID14	SVID18	SVID29	SVID30	SVID31
Doppler frequency range (Hz)	87,900	87,900	87,900	87,900	87,900	87,900
Maximum $C/N_{0}$	36.25	36.5	45.5	36.75	45.5	45
Minimum $C/N_{0}$	35	35.25	44.5	35.75	44.75	44.25
Average $C/N_{0}$	35.71	36.01	44.93	36.18	45.12	44.67

**Table 2:** Statistical table of Experiment 1 Results.

Experiment 1	SVID9	SVID14	SVID18	SVID29	SVID30	SVID31
Doppler frequency range (Hz)	20,950	20,940	20,980	20,940	20,990	20,990
Maximum $C/N_{0}$	36.25	40.25	43	42.5	37.75	37.25
Minimum $C/N_{0}$	35.25	39.25	42.25	41.75	36	36.25
Average $C/N_{0}$	35.74	39.75	42.62	42.13	36.89	36.7

## Data Availability

The data used in this research is not publicly available.
